# Multimodal optical analysis discriminates freshly extracted human sample of gliomas, metastases and meningiomas from their appropriate controls

**DOI:** 10.1038/srep41724

**Published:** 2017-02-02

**Authors:** Marc Zanello, Fanny Poulon, Johan Pallud, Pascale Varlet, H. Hamzeh, Georges Abi Lahoud, Felipe Andreiuolo, Ali Ibrahim, Mélanie Pages, Fabrice Chretien, Federico Di Rocco, Edouard Dezamis, François Nataf, Baris Turak, Bertrand Devaux, Darine Abi Haidar

**Affiliations:** 1 IMNC Laboratory, UMR 8165-CNRS/IN2P3, Paris-Saclay university, 91405 Orsay, France; 2 Neurosurgery Department, Sainte-Anne Hospital, France; 3 Paris Descartes University, Paris, France; 4 Neuropathology Department, Sainte-Anne Hospital, France; 5 Center of Advanced European Studies and Research (caesar), 53175 Bonn, Germany; 6 Neurosurgery Department, Lyon Hospital, France; 7 Paris Diderot University, Sorbonne Paris Cité, F-75013, Paris, France

## Abstract

Delineating tumor margins as accurately as possible is of primordial importance in surgical oncology: extent of resection is associated with survival but respect of healthy surrounding tissue is necessary for preserved quality of life. The real-time analysis of the endogeneous fluorescence signal of brain tissues is a promising tool for defining margins of brain tumors. The present study aims to demonstrate the feasibility of multimodal optical analysis to discriminate fresh samples of gliomas, metastases and meningiomas from their appropriate controls. Tumor samples were studied on an optical fibered endoscope using spectral and fluorescence lifetime analysis and then on a multimodal set-up for acquiring spectral, one and two-photon fluorescence images, second harmonic generation signals and two-photon fluorescence lifetime datasets. The obtained data allowed us to differentiate healthy samples from tumor samples. These results confirmed the possible clinical relevance of this real-time multimodal optical analysis. This technique can be easily applied to neurosurgical procedures for a better delineation of surgical margins.

Surgical resection, whenever feasible, remains the first line of therapy to treat central nervous system tumors^1^,^2^. The extent of resection is a major prognostic factor, whatever the histopathological subtype^3^. Although maximal resection is required, preserving surrounding healthy brain areas is warranted to equilibrate the onco-functional balance: improving the outcomes through maximal tumor removal and preserving the postoperative quality of life. The identification of the tumor limits, in case of gliomas, metastases and meningiomas may be difficult intraoperatively. Intraoperative microscopy, intraoperative ultrasonography, or intraoperative MRI are insufficient to resolve tissue microstructure and discriminate between tumor induced tissue alterations and surgically induced changes, such as contusion, ischemia or edema^4^,^5^,^6^.

The most notable achievement concerning optical imaging for brain tumor margin delineation was realized by Stummer *et al*. with the use of external dye, δ-aminolevulinic acid (ALA)^7^. Clinical studies have shown an increased extent of resection and improved overall survival using ALA^8^,^9^, however, its sensitivity can be improved, especially in infiltrative areas^10^,^11^. Moreover, any dye has contraindications and side effects. Our group tries to overcome these limits by analyzing human tissue endogenous fluorescence. First attempts of label-free optical imaging were made during 1990’s and active research is still currently underway in this field with no major clinical translation^12^,^13^,^14^,^15^,^16^. Meanwhile, nonlinear microscopy technique has emerged over the last 20 years^17^,^18^. It differs from classical confocal microscopy by its ability to use several endogeneous contrasts, especially second harmonic generation and two-photon fluorescence without introducing exogenous dye. The near infrared excitation source allows a deeper penetration and a lower level of photodamage compared with confocal microscopy, which helps to preserve tissue. Autofluorescence analysis is difficult primarily due to the low signal to noise ratio: the combination of multiple modalities could help distinguish tumor margins more precicely than techniques that offer a unique modality^19^,^20^,^21^. In this work we implement spectral and lifetime fluorescence in spatial imaging. This quantitative information provides additional information on the physicochemical environment and molecular interaction.

The present study investigated the ability of visible and nonlinear optical imaging techniques to discriminate between tumor and control healthy tissues. For this purpose, metastasis, glioma and meningioma samples were compared to their appropriate controls: healthy brain samples and dura mater samples, respectively. In this study, thin human samples, freshly extracted, are analyzed to be as close as possible to clinical conditions. We made qualitative and quantitative measurements on these different sample groups. This comparative study was made on two set-ups: endoscopic fibered set-up and multimodal microscope. With the optical fibered endoscope we acquired spectroscopic and lifetime measurements on freshly extracted human samples as a first step before *in vivo* clinical measurements. It allows excitation with two different visible wavelengths, 375 and 405 nm. These wavelengths are well known^22^,^23^ to excite different endogenous molecules, like reduced nicotinamide adenine dinucleotide (NADH), flavins (FAD), lipopigment, porphyrins. Our team is currently working on a new surgical tool that allows performing nonlinear optical analysis of endogenous fluorescence in real time during neurosurgical procedures. To further enhance the specificity and sensitivity of the optical analyses, four different contrasts will be studied: (1) one and two-photon spectral detection, (2) time domain measurement and two-photon Fluorescence Lifetime Imaging, (3) second harmonic generation imaging and (4) one and two-photon fluorescence imaging. The aim is to help neurosurgeons to realize maximal resection of brain tumors based on the endogenous contrast between the fluorescence of tumor tissue and healthy surrounding tissue.

As a first step before this fibered multimodal optical surgical tool, we have chosen to use a multimodal bench top microscope. It allows us to (i) test the safety of multimodal excitation on fresh human samples, (ii) obtain the four different contrasts previously discussed, (iii) compared fluorescence images with gold standard histopathology and (iv) discriminate potential differences between the different samples (glioma, metastasis, meningioma, and healthy cortex).

## Results

### Samples

Clinical and histopathological characteristics are summarized in Table 1. Fifty-four samples (tumor and control samples) were analyzed. Thirty-eight fresh human tumor samples from 38 patients (21 men, 17 women, mean age at diagnosis 58.3 ± 12.1 years) have been analyzed on the endoscopic fibered set-up and ten of these 38 samples (26%) have also been analyzed on the nonlinear microscopic set-up. This cohort was divided into two parts: intra-axial tumor part, subdivided in Glioma group (n = 16) and Metastasis group (n = 14), and extra-axial tumor part, Meningioma group (n = 8). Each part was compared to appropriate controls: healthy cortex samples (n = 11) for intra-axial tumor part, named Control Group and healthy dura mater samples (n = 5) for extra-axial tumor part, named Meningioma Control Group.

No significant difference in tissue alteration was noticed between the samples analyzed with the optical endoscope only and the samples analyzed on both set-ups.

### Fibered set-up results

#### Spectral measurements of endogenous fluorescence

**Figure 1:**
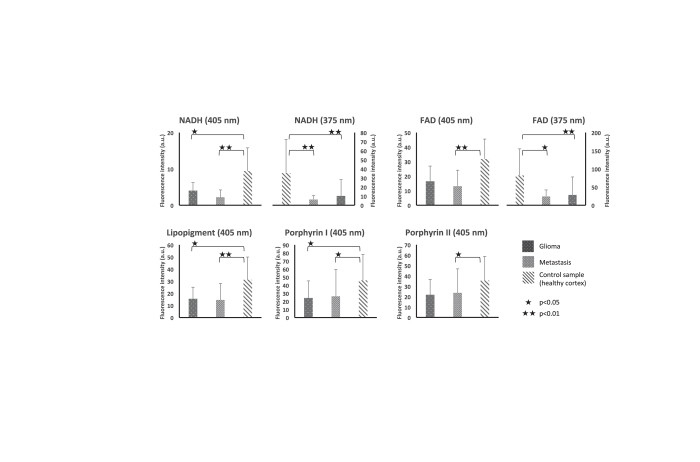
Results of spectroscopic endogenous fluorescence measurements for intra-axial tumor part. Fluorescence intensity of NADH and FAD when exciting with 405 and 375 nm for Glioma, metastasis and control samples. Results for Lipopigment, porphyrin I and II are presented for 405 nm excitation wavelength. ^★★^Under a bar denote statistically significant difference (p < 0.01).

As shown in Fig. 1, glioma group autofluorescence is almost two times lower than control group autofluorescence. At 405 nm excitation wavelength and at 375 nm excitation wavelength, fluorescence intensity from glioma tissue was lower for all investigated endogenous molecules than the control group. This difference was significant for NADH (p = 0.018), lipopigment (p = 0.038), and porphyrin I (p = 0.048) whereas a trend existed concerning FAD (p = 0.054), and porphyrin II (p = 0.126) without reaching statistical significance. At 375 nm excitation wavelength, fluorescence emission from glioma tissue was also reduced for NADH (p = 0.007) and FAD (p = 0.005).

Metastasis group emitted significantly less autofluorescence than control group. At 405 nm and at 375 nm excitation wavelengths, fluorescence intensity was significantly reduced at all investigated emission wavelengths compared to control group. With 405 excitation wavelength, fluorescence emission of each molecules from glioma samples are lower than control: NADH (p > 0.001), FAD (p = 0.002), lipopigment (p = 0.006), porphyrin I (p = 0.033) and porphyrin II (p = 0.029). At 375 nm excitation wavelength, fluorescence intensity was also reduced for NADH (p = 0.009) and FAD (p = 0.012).

Glioma and Metastasis groups did not exhibit significant differences. However, fluorescence intensity was reduced for NADH in Metastasis group compared to Glioma group with 375 nm and 405 nm excitation wavelengths without reaching statistical significance.

**Figure 2:**
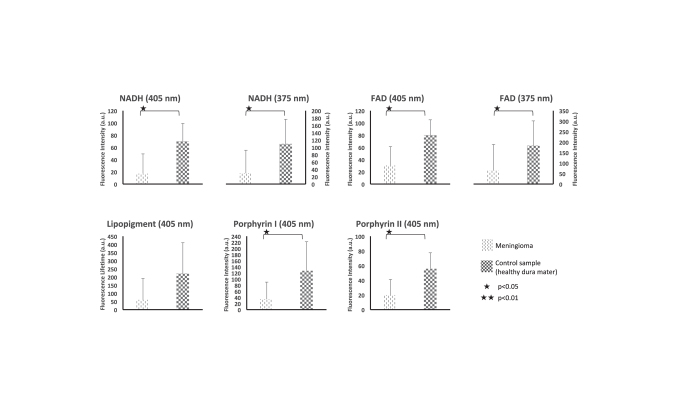
Results of spectroscopic endogenous fluorescence measurements for extra-axial tumor part. Fluorescence lifetime of NADH and FAD when exciting with 405 and 375 nm from meningioma and control samples. Results for Lipopigment, porphyrin I and II are presented for 405 nm excitation wavelength.

Meningioma group presented a 4-fold decrease in autofluorescence compared to meningioma control group (Fig. 2). At 405 nm excitation wavelength, fluorescence intensity was significantly reduced at four investigated endogenous molecules compared to meningioma control group: NADH, FAD, porphyrin I and porphyrin II (p = 0.028, p = 0.028, p = 0.019, and p = 0.019, respectively). At 375 nm emission wavelength, fluorescence intensity was reduced for both NADH and FAD (p = 0.019, and p = 0.028, respectively).

#### Lifetime measurements of endogenous fluorescence

Lifetime values of glioma, metastasis, meningioma and respective control group using 375 and 405 nm excitation wavelength are resumed in Table 2.

**Figure 3:**
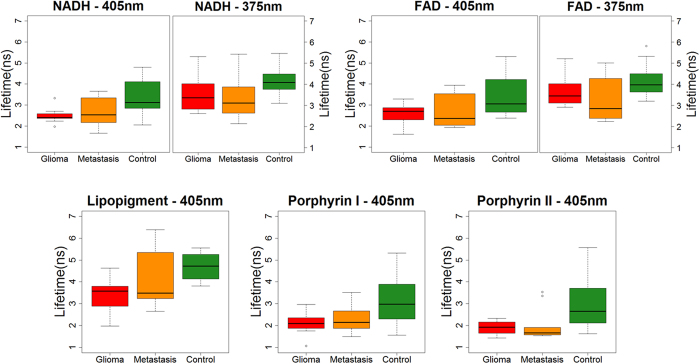
Box plot of endogenous fluorescence lifetime measurements for intra-axial tumor part. Lifetime variation of NADH and FAD when exciting with 405 and 375 nm from Glioma, metastasis and control samples. Results for Lipopigment, porphyrin I and II are presented for 405 nm excitation wavelength. * and ** denote statistically significant difference (p < 0.05 and p < 0.01, respectively).

Using 405 nm excitation wavelength, fluorescence lifetime measurements were significantly shorter in glioma group than in control group for NADH (p = 0.008), FAD (p = 0.035) and lipopigment (p = 0.035). As shown in Fig. 3, no significant difference existed using 375 nm excitation wavelength.

Concerning metastasis group, the same trend as glioma group is observed as shown in Fig. 3. Fluorescence lifetime values were significantly shorter in metastasis group than in control group for NADH (p = 0.009), FAD (p = 0.026) and lipopigment (p = 0.040). No significant difference existed at 375 nm excitation wavelength.

**Figure 4:**
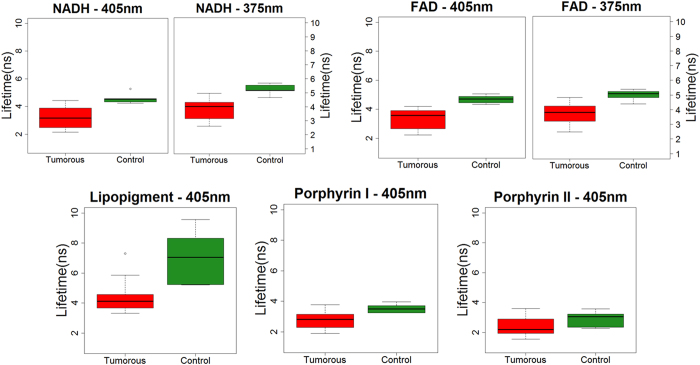
Box plot of Endogenous Fluorescence lifetime measurements for extra-axial tumor part. Lifetime variation of NADH and FAD when exciting with 405 and 375 nm from tumorous and control tissue. Results for Lipopigment, porphyrin I and II are presented for 405 nm excitation wavelength. * and ** denote statistically significant difference (p < 0.05 and p < 0.01, respectively).

With 405 and 375 nm excitation wavelengths, fluorescence lifetime values were significantly reduced in meningioma group compared to meningioma control group. Figure 4 present this tendency.

### Results on multimodal microscopic imaging set-up

#### Choice of the excitation wavelength

A specific spectral study was accomplished to define the best excitation wavelength able to excite endogenous fluorescence as well as second harmonic generation signal. For this purpose we made an excitation-emission matrix, thanks to the tunable Ti:sapphire lasers (from 690 to 1040 nm). This study reveals that 810 nm was best suited to excite endogenous fluorescence, whereas 890 nm was the adequate wavelength to excite fluorescence as well as second harmonic generation signal and confirmed our previous results^22^. These two wavelengths was used as excitation wavelength for our study.

#### Multimodal nonlinear analysis of endogenous fluorescence under nonlinear excitation

**Figure 5:**
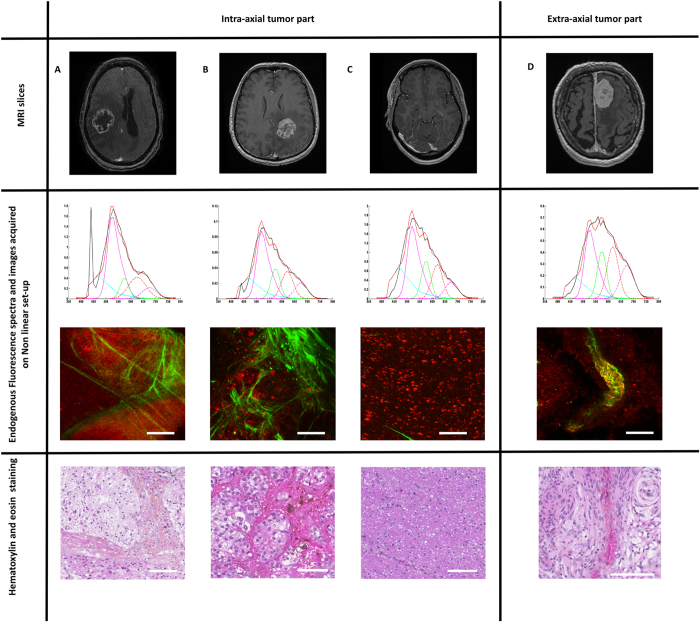
Multimodal analysis of intra and extra- axial tumor analysis of glioma (**A**) and metastasis (**B**) samples versus control samples (**C**) and meningioma control sample (**D**). From top to bottom: Axial MR slice on contrast enhanced T1-weighted sequence. Spectra at 890 nm excitation wavelength from nonlinear set-up showing a clear peak of SHG at 445 nm. Image from nonlinear set-up showing SHG signal in green and fluorescence in red. Hematoxylin-Eosin staining Images. Scale bars: 100 μm. Spectra analysis: X-axis: emission wavelength (nm) and Y-axis: Fluorescence intensity (a.u.); Fitted by Matlab software: blue, purple and green line represent respectively NADH, FAD and lipopigment fluorescence emission spectra. Red and purple dot lines for porphyrin I and porphyrin II.

Figure 5 illustrates the results of this multimodal analysis of intra and extra- axial tumor analysis.

#### A-Glioma group

Using two-photon excitation, at 810 nm excitation wavelength, glioma samples showed a peak centered at 540 nm and a second peak at 660 nm. At 890 nm excitation wavelength, besides the two described peaks, second harmonic generation signal was visible at 445 nm. At 810 nm excitation wavelength, FLIM study found out two components with lifetimes values of 2.40 ns and 0.40 ns, always longer than the values of control group.

#### B-Metastasis group

At 810 nm excitation wavelength, metastasis samples exhibited one peak centered at 540 nm. At 890 nm excitation wavelength, the same peak centered at 540 nm was observed as well as second harmonic generation signal was detected around 445 nm. At 810 nm excitation wavelength, FLIM study found out two components with lifetime’s values of 2.57 ns and 0.50 ns, once again the values increased compared to control group.

#### C-Control group

At 810 nm excitation wavelength, control samples showed a peak centered at 520 nm. At 890 nm excitation wavelength, the same peak was observable. At 810 nm excitation wavelength, FLIM study showed two components with lifetimes values of 2.10 ns and 0.35 ns.

#### D-Meningioma group

At 810 nm excitation wavelength, meningioma samples emitted three peaks centered at 520 nm, 580 nm and 640 nm with a broad fluorescence emission. At 890 nm excitation wavelength, a peak at 445 nm was visible corresponding to second harmonic generation signal. At 810 nm excitation wavelength, FLIM study showed three components with lifetimes values of 2.30 ns, 1.8 ns and 0.40 ns.

Using one photon excitation, all tumors spectra were red shifted and presented lower fluorescence intensity than control samples. These statements were less visible with two-photon excitation. Spectrum shape and intensity were quite different for each tissue type. Extra-axial and intra-axial tumors strongly differentiate with a broader spectrum in extra-axial due to a higher contribution of porphyrins and chlorins, a difference is also noticeable in the three types of intra-axial tissues, glioma appears narrower in the principal peak, explained by a very low lipopigment signal, compared to metastasis and control tissue.

#### Comparison with gold standard histopathology

Figure 5 represents illustrative cases and we observed variability in spectral acquisition from a sample to another. Two-photon images revealed the same structures as than gold standard H-E images. The second harmonic generation signal, in green, delimitated lobules in metastasis and necrosis in glioma whereas it was absent from the control sample. It corresponded to the spectral acquisition made at 890 nm excitation wavelength. Autofluorescence signal, in red, underlined cell cytoplasm and, for instance, showed the lobular arrangement in the meningioma sample.

Image from nonlinear set-up showing SHG signal in green corresponding to collagen fibers and two distinct zones of red fluorescence, one pale corresponding to necrosis and the second intense corresponding to glioma cells. Hematoxylin-Eosin staining showing necrosis and collagen fibers.

## Discussion and Conclusion

We have, for the first time, carried out a multimodal optical analysis in a cohort of 58 fresh human brain tumors samples compared to their appropriate control samples. We showed that: (i) glioma, metastasis and meningioma emitted significantly lower fluorescence than their controls at 405 and 375 nm excitation wavelengths; (ii) at 405 nm excitation wavelength, lifetime values of glioma, metastasis and meningioma were shorter than their controls and at 375 nm we did not find any difference; (iii) multimodal nonlinear analysis of human brain samples provided new insights in the tissue architecture upon analyzing FLIM, two-photon fluorescence and second harmonic generation signal. Altogether, those results suggest multimodal optical analysis as a promising tool to help the intraoperative identification of tumor margins.

Our study confirmed that fluorescence emission was significantly reduced in tumor tissue as compared to healthy tissue in accordance with previous reports^12^,^13^,^14^,^16^,^24^,^25^,^26^,^27^. The autofluorescence decrease we observed in tumor tissues had been ascribed to reduction of NADH amount in tumor cells due to the Warburg effect, the increased metabolization, almost tenfold, of glucose to lactate by neoplastic tissue compared with healthy tissue in aerobic condition^28^,^29^. However, this point is still a matter of debate with a recent review quoting a possible increase of NADH with mitochondrial dysfunction in tumor^30^. It might be possible that tumor necrosis (up to 87% of glioblastoma in the present series), tumor hyperperfusion or neoangiogenesis explained, at least in part, the observed lower emission fluorescence^31^,^32^. Previous studies reported a shift in the emission wavelength between the different tissue types^16^,^25^. In the present series, we observed a redshift using one photon excitation. This trend was less evident with two-photon excitation but the main peak was centered at 540 nm for metastasis and glioma versus 520 nm for the control samples.

Even if analysis of fluorescence intensity seems promising to discriminate tumor infiltration from normal zones, some drawbacks must be taken into account: spectral shape was quite variable, blood contamination during surgery can drastically decrease the collected signal. Distinguishing tumor borders from healthy tissue should be more difficult than differentiating solid tumor and healthy tissue. In this way, multimodal analysis seems mandatory^33^.

Fluorescence lifetime is a sensitive technique for recording low-level signals with high precision^34^. To our knowledge, our lifetime measurements are the only ones using 375 and 405 nm excitation wavelengths. The absence of any previous work using the same excitation wavelengths makes harder comparison with literature. Lifetime values that we obtained are in accordance with general review on this topic but not with the previous work on human brain samples^12^,^24^,^35^,^36^,^37^,^38^. However, the values remained in the same order of magnitude. For instance, Yong *et al*. used a 337 nm excitation wavelength and found out lifetime values at 390 nm emission wavelength equal to 1.27 ns for cerebral cortex; 2.3 ns for normal white matter; 1.4 ns for low grade glioma; 1.4 for high grade glioma and 2.0 ns for high grade glioma with necrotic change. At 440 nm and 460 nm, emission wavelengths closer to the studied emission wavelength in this paper, this trend was less evident. In our multivariate analysis, 470 nm corresponding to NADH, 520 nm corresponding to flavins and 580 nm corresponding to lipopigments had significantly longer lifetime values in the control group than tumor groups using 405 nm as excitation wavelength. This is in accordance with a previous work by our team on rats tissues and literature^23^,^39^. NADH lower lifetime in neoplastic tissue can be explained by change in NADH bound/free ratio or changes in the distribution of NADH enzyme binding sites^39^,^40^.

Our literature review found out some works of multimodal nonlinear optical microspectroscopy on brain or nervous system^41^,^42^ but only one multiphoton analysis of human brain samples was performed with 750 nm excitation wavelength using the DermaInspect set-up^43^. FLIM results ranged from 1.4 ns (brain parenchyma sample) to 2.1 ns (glioblastoma sample)^43^. Correlation between gold standard histopathology was made only on rat samples and no second harmonic generation study was performed. We worked on a dedicated set-up for nonlinear analysis and we recorded four different types of signals: second harmonic generation, FLIM, one and two-photon fluorescence imaging. This preliminary study confirmed the potential of nonlinear microscopy for human brain investigation: mosaics composed of second harmonic generation signal and fluorescence signal at 890 nm excitation wavelength are presented in Fig. 5. Second harmonic generation signal revealed collagen fibers, present in necrosis for example. Fluorescence showed multiple focal points, corresponding to cell cytoplasm or mitochondria. A pathologist made a comparison between nonlinear images and HE slides. Matching mosaics and HE slides was possible in the ten cases. No macroscopic tissue alteration was found out after two-photon excitation. Despite the limits of such macroscopic analysis, this results was in accordance with previous results^44^,^45^. Our FLIM measurements were in accordance with a previous study performed on fixed meningioma samples^22^. Two-photon FLIM measurement differed from the results of endoscopic visible set-up due to several reasons: (1) the excitation wavelength and the focal volume (due to the different setup configuration) were not the same and by consequence the lifetime could differ; (2) the absorption effective section of the various molecules was different under two-photon excitation; (3) under two-photon excitation, we collected the global emission spectra without any selective filter in front of detectors as in the endoscopic set-up.

*Ex vivo* condition was the major limitation of this study. However, the whole process took less than two hours ensuring the fresh condition of our samples^46^ and this delay was essentially due to the transportation of samples. Even if we presented the largest cohort of *ex vivo* fresh human brain samples to date, another limit was the definition of our groups: gliomas vary by subtypes and grade and metastases vary by their primary. Such subgroup analyses were not possible to maintain robust statistical analyses. The same consideration remained true for the effects of oncological treatment on fluorescence signal. Finally the control group (healthy brain tissue) used in the study could not have same fluorescence/optical characteristics as the normal brain tissue surrounding the tumor but this control group definition ensured that our control samples were healthy, without any tumoral infiltration. Moreover, the small number of samples analyzed on the multimodal microscopic setup precluded any relevant statistical calculations.

These results represent the first step in producing optical signatures from human brain tissue with multimodal analyses using one and two-photon excitation. This emerging database needs to be widened. A nonlinear endomicroscope adapted to the intraoperative condition is under development by our group. Given that the aim of this surgical tool is to distinguish infiltrated and healthy tissue, it seems mandatory to combine second harmonic generation signal, spectral analysis, lifetime values and two-photon fluorescence to detect infiltrated tissue and not only solid tumor. Multimodal analysis may potentially help differentiating tumor infiltration from healthy zones, allowing the improvement of the quality of life and the survival of patients harboring a central nervous system tumor. Such nonlinear endomicroscopy presents obvious advantages: (1) easy to use in the operating room; (2) fast time acquisitions; (3) and low cost^13^. Moreover, subcellular investigation of human brain can help current research on many topics such as age related diseases or psychiatric diseases.

## Material and Methods

### Samples

The inclusion criteria for this monocentric prospective collection of fresh samples were: (1) adult patients; (2) newly diagnosed central nervous system tumor at the Sainte-Anne Hospital (Paris, France); (3) available fresh samples to be analyzed on optical set-ups in addition to the routine histopathology protocols; (4) selection by a senior neuropathologist of representative and homogeneous samples (meningioma, glioma, metastasis, and control). The institutional review board of the Sainte-Anne Hospital center – University Paris Descartes approved the study protocol (number SC3227), all the following methods were performed in accordance with the relevant guidelines and regulations issue in this protocol. Moreover informed consents were obtained from all the samples coming from human subjects.

Sample size varied from 32 mm^3^ (4 × 4 × 2 mm) to 750 mm^3^ (15 × 10 × 5 mm). They were maintained in normal saline solution to avoid desiccation, in a temperature-controlled dark room dedicated to optical imaging. They were cut with a scalpel to obtain a planar surface. The sample was studied first on a visible set-up then on a nonlinear multimodal set-up. The whole process took less than 120 min. The fresh samples were then fixed with 4% paraformaldehyde, embedded in paraffin and stained with Hematoxylin-Eosin then digitized using Digital Slide Scanner NanoZoomer 2.0 (Hamamatsu Photonics K.K, Hamamatsu, Japan). Detailed clinical data including age at diagnosis, past medical history and histopathological data including presence of mitosis, necrosis, neoangiogenesis and immunohistochemical analysis were recorded.

We examined non-tumor brain parenchyma fresh samples as control samples for intraparenchymal tumor samples (glioma and metastasis) and dura mater samples for meningioma samples. The non-tumor brain parenchyma samples (n = 11, the so-called control group) provided from patients operated for a drug-resistant temporal epilepsy during the same period. Dura mater control samples (n = 5, the so-called meningioma control group) provided from dural boundaries far from meningioma resection.

A senior neuropathologist performed a central review of definitive Hematoxylin-Eosin staining of the study’s samples, without prior knowledge of the two-photon analysis status (performed or not). Detected tissue alteration were reported to the investigators.

### Optical endoscope architecture

**Figure 6:**
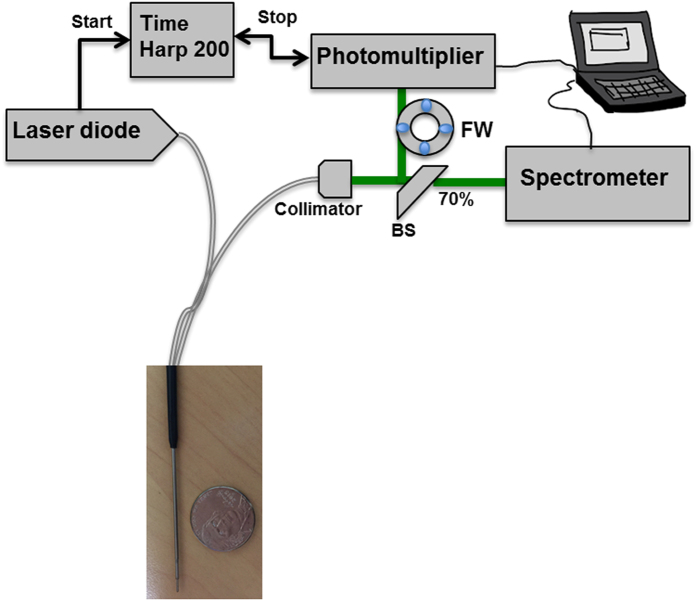
Optical fibered endoscope set up. Spectral and lifetime measurements were acquired using this set up and under 405 and 375 nm excitation wavelength. Two fibered was used for excitation and fluorescence collection.

Two pulsed diode lasers, one emitting at 405 nm and the other one at 375 nm, from PicoQuant (GmbH, Berlin, Germany) were used as laser sources. The diode 375, FWHM 45 ps and 405 nm, FWHM 60 ps were used. The same “Sepia” driver controlled the diodes. Repetition frequency can be set between 2.5 and 40 MHz. Repetition frequency used for this study is 40 MHz. Excitation and collection were acquired thanks to a bi-fibered configuration. The fibres used for tissue excitation and collection had a core diameter of 200 μm and 365 μm, respectively, with a numerical aperture of 0.22. The spatial resolution was of 500 μm. A beam Splitter divided and sent the collected fluorescence into two detectors: 70% of the signal toward a computer controlled cooled spectrometer (Ocean optics QP600-1-UV-VIS) for spectroscopic analysis and 30% toward a Photomultiplier Tube a Photomultiplier Tube (PMT) (PMA-182 NM, PicoQuant GmbH, Berlin, Germany) for lifetime measurement. The synchronization output signal from the diode driver and the start signal from the PMT were connected to their respective channels on the data acquisition board Time-Correlated Single Photon Counting (TCSPC) (TimeHarp 200, PicoQuant GmbH, Berlin, Germany). A filter wheel was used to select the spectral emission band during lifetime measurement. Five filters were used when exciting with 405 nm (450 ± 10 nm, 520 ± 10 nm, 550 ± 30 nm, 620 ± 10 nm and 680 ± 10 nm). Using theses filters we can respectively detect the emitted fluorescence from NADH, FAD, lipopigments, porphyrin I and porphyrin II. Two filters are used when exciting with 375 nm (450 ± 10 nm and 520 ± 10 nm) for NADH and FAD (Fig. 6). Each lifetime measurement lasted 2 seconds. Data were adjusted by a mono-exponential fit via fluofit software (PicoQuant, GmbH, Berlin, Germany) to recover the lifetimes from the measured fluorescence decays.

Spectral acquisition was accomplished for several longitudinal lines of each sample thanks to a specific mechanical support mounted on a motorized microtranslator stage (Thorlabs, Newton, USA)^23^ and it lasted 5 to 10 minutes per sample.

The spectral measurements were processed using a homemade Matlab program, as previously described^23^. The same five fluorophores were considered: reduced Nicotinamide adenine dinucleotide (NADH), flavins (FAD), lipopigments, porphyrin I and porphyrin II. Data were adjusted by a mono-exponential fit via FluoFit software (PicoQuant, GmbH, Berlin, Germany) to recover the lifetimes from the measured fluorescence decays.

### Microscopic multimodal setup: Confocal, two-photon and FLIM imaging of endogenous fluorescence

A Mai Tai DeepSee Ti:Sapphire laser source (Mai Tai DeepSee, Spectra-Physics, Santa Clara, USA) with automated dispersion compensation. The average power is around 2.4 W at 800 nm excitation wavelength. It’s tunable from 690 to 1040 nm. The repetition rate of the laser source was 80 MHz and the output pulse duration was around 70 fs. This laser is combined to a TCS SP8 MP confocal microscope from Leica Microsystems (Leica Microsystems, Wetzlar, Germany). This set up was used for confocal, two-photon and fluorescence lifetime (FLIM) imaging of samples. The presence of two additional filters (FF01-448/20-25 and FF01-520/35-25, Semrock, New York, USA) on the dichroic cube set the detection band for each path toward one of the two external hybrids.

This optical set-up recorded four different optical signals on the Regions Of Interest (ROI): (1) one and two-photon Spectral analysis, (2) two-photon FLIM measurement, (3) second harmonic generation imaging and (4) Fluorescence imaging under one and two-photon excitation.

Analyses were performed using the dedicated Leica software (Leica Microsystems, Wetzlar, Germany). Fluorescence and second harmonic generation images processing were made using the dedicated Leica software as well as Matlab and imageJ. For FLIM measurements, three positions at least per sample were analyzed using 810 and 890 nm excitation wavelengths. The data were collected and analyzed via the software Symphotime (PicoQuant, GmbH, Berlin, Germany). On each FLIM image, twelve ROI were selected on the different structures and fitted by a mono or bi exponential decay to extract the fluorescence lifetime.

### Statistical analyses

Fluorescence intensity and lifetime results were evaluated using a one-way analysis of variances (ANOVA). If the ANOVA was statistically significant, a post-hoc Wilcoxon test was performed. A probability value (p) < 0.05 was considered statistically significant. All statistical analyses were performed using JMP software (version 11.0.0, SAS Institute Inc).

## Additional Information

**How to cite this article:** Zanello, M. *et al*. Multimodal optical analysis discriminates freshly extracted human sample of gliomas, metastases and meningiomas from their appropriate controls. *Sci. Rep.* **7**, 41724; doi: 10.1038/srep41724 (2017).

**Publisher's note:** Springer Nature remains neutral with regard to jurisdictional claims in published maps and institutional affiliations.

## Figures and Tables

**Table 1:** Clinical and Histopathological Characteristics of our human samples series.

Group	Glioma Group		Metastasis Group		Control Group		Meningioma Group		Meningioma Control Group	
Parameter	(n = 16)		(n = 14)		(n = 11)		(n = 8)		(n = 5)	
	No.	%	No.	%	No.	%	No.	%	No.	%
**Clinical findings**										
Mean age at diagnosis, years	57.7	—	57.2	—	40.1	—	61.4	—	72.6	—
Range	26.2–77.4	—	39.8–74.4	—	23.9–61.9	—	53.2–72.4	—	58.6–92.3	—
Gender										
Male	11	69	6	43	5	45	2	25	1	33
Female	5	31	8	57	6	55	6	75	2	67
Previous treatment on Central Nervous system (Surgery and/or Radiotherapy and/or Chemotherapy)	2	13	0	0	0	0	2	25	0	0
Two-photon Multimodal Imaging										
Yes	4	25	3	21	3	27	3	38	0	0
No	12	75	11	79	8	63	5	62	5	100
**Histopathological Findings**										
Necrosis										
Yes	12	75	12	86	0	0	2	25	0	0
No	4	25	2	14	11	100	6	75	5	100
**Angiogenesis**										
Yes	13	81	1	7	0	0	1	13	0	0
No	3	19	13	93	11	100	7	87	5	100
Mean Proliferation rate (%)	21		—*		—		5		—	
Range	10–40		—		—		1–12		—	

^*^Only one sample had a calculated proliferation rate.

**Table 2:** Fluorescence Lifetime measurements of our human samples series.

Lifetime values (ns)	Glioma Group	Metastasis Group	Control Group	Meningioma Group	Meningioma control Group
	(n = 16)	(n = 14)	(n = 11)	(n = 8)	(n = 5)
Parameter	Mean (SD)	Mean (SD)	Mean (SD)	Mean (SD)	Mean (SD)
**λ**_**exc**_** = 375 nm**					
NADH	4.01 (1.29)	4.05 (1.02)	3.41 (0.80)	3.94 (0.58)	5.30 (0.30)
FAD	3.88 (1.18)	4.29 (0.81)	3.87 (0.56)	3.90 (0.69)	5.22 (0.27)
**λ**_**exc**_** = 405 nm**					
NADH	2.55 (0.61)	2.71 (0.51)	3.38 (0.62)	3.22 (0.73)	4.58 (0.98)
FAD	2.71 (0.64)	2.71 (0.53)	3.29 (0.55)	3.27 (0.67)	4.83 (0.87)
Lipopigment	3.80 (1.18)	3.66 (0.80)	5.21 (2.72)	3.86 (0.88)	9.58 (5.16)
Porphyrin I	2.57 (0.88)	3.00 (1.17)	2.92 (1.12)	2.60 (0.46)	4.00 (0.88)
Porphyrin II	2.41 (1.19)	2.53 (1.16)	2.96 (0.92)	1.91 (0.36)	3.25 (0.77)
